# Intestinal Lymphangioma With Extensive Secondary Lymphangiectasia Causing Life‐Threatening Hemorrhage: First Case Requiring Small Bowel Transplantation

**DOI:** 10.1155/crip/6663208

**Published:** 2026-09-12

**Authors:** Nasibeh Sargazi Moghadaam, Bita Geramizadeh, Zahra Hajizadeh, Hamed Nikoupour, Sara Farifteh

**Affiliations:** ^1^ Faculty of Medicine, Shiraz University of Medical Sciences, Shiraz, Iran, sums.ac.ir; ^2^ Shiraz Transplant Research Center (STRC), Shiraz University of Medical Sciences, Shiraz, Iran, sums.ac.ir; ^3^ Department of Pathology, Shiraz University of Medical Sciences, Shiraz, Iran, sums.ac.ir; ^4^ Shiraz Transplant Center, Abu-Ali Sina Hospital, Shiraz University of Medical Sciences, Shiraz, Iran, sums.ac.ir

**Keywords:** gastrointestinal hemorrhage, intestinal lymphangioma, mesenteric lymphadenopathy, secondary lymphangiectasia, small bowel transplantation

## Abstract

**Background:**

Intestinal lymphangiectasia is a rare disorder characterized by dilated lymphatic vessels in the gastrointestinal tract, typically presenting with protein‐losing enteropathy. Adult‐onset cases with severe gastrointestinal bleeding are exceptionally rare.

**Case Presentation:**

A 40‐year‐old male presented with severe recurrent gastrointestinal bleeding and iron‐deficiency anemia. Double‐balloon enteroscopy revealed diffuse jejunal infiltration with white villi throughout the jejunum, consistent with lymphatic dilation. CT imaging showed mesenteric lymphadenopathy. Technetium‐99m–labeled red blood cell scintigraphy demonstrated active bleeding within the superior mesenteric artery distribution. Histopathological examination confirmed intestinal lymphangioma with extensive secondary lymphangiectasia. Despite conservative management, refractory bleeding requiring over 20 units of packed red blood cells necessitated small bowel transplantation with subtotal colectomy. Pathological examination of the resected specimen revealed extensive lymphangiectasia affecting the small intestine, colon, appendix, and gallbladder. Notably, all mesenteric lymph nodes showed only reactive hyperplasia, excluding a lymphoproliferative disorder. The patient developed postoperative complications, including extensive venous thrombosis, empyema, and multiorgan failure, succumbing on postoperative Day 20.

**Conclusion:**

This represents the first reported case of intestinal lymphangioma with extensive secondary lymphangiectasia requiring small bowel transplantation. The atypical presentation without classic protein‐losing enteropathy features, multiorgan gastrointestinal involvement, and fatal outcome highlight the variable clinical spectrum and potential severity of this rare condition.

## 1. Introduction

Intestinal lymphangiectasia (IL) is characterized by abnormally dilated lymphatic vessels, classified as primary or secondary [1, 2]. Primary intestinal lymphangiectasia (PIL), described by Waldmann in 1961, is a rare congenital disorder (prevalence less than 1 in 100,000) manifesting as protein‐losing enteropathy: hypoalbuminemia, hypogammaglobulinemia, and lymphopenia [3–5]. Secondary lymphangiectasia results from lymphatic obstruction due to malignancies, inflammatory diseases, infections, or cardiovascular disorders [2, 6, 7].

Intestinal lymphangioma is a benign hamartomatous malformation with true neoplastic proliferation of lymphatic tissue, distinct from simple vessel dilatation [1–3]. Lymphangiomas can cause secondary lymphangiectasia through mass effect or lymphatic drainage disruption [8, 9]. Typical PIL presentation includes edema, ascites, and diarrhea with characteristic laboratory abnormalities [1, 5]. Lymphangioma usually presents with abdominal pain or obstruction; gastrointestinal bleeding is exceptionally rare [10–13].

We present the first case of intestinal lymphangioma causing extensive secondary lymphangiectasia treated with small bowel transplantation. Unique features include life‐threatening gastrointestinal bleeding requiring more than 20 units of blood without protein‐losing enteropathy, absence of typical laboratory abnormalities, multiorgan involvement (small bowel, colon, appendix, and gallbladder), and fatal postoperative complications. This case highlights the severity of extensive lymphatic disease and the importance of identifying lymphangioma as the underlying pathology.

## 2. Case Presentation

A 40‐year‐old male was admitted with gastrointestinal bleeding, iron‐deficiency anemia, and abdominal pain. The patient provided written informed consent for the publication of this case report and associated images. In August 2021, contrast‐enhanced CT of the abdomen and pelvis showed multiple hypodense lymphadenopathies in the mesentery of the small intestine, suggesting a possible lymphoproliferative disorder. Mild ascites were observed in the pelvic region, with no dilatation of the bowel loops.

In light of these findings, a double‐balloon enteroscopy was performed, which showed diffuse infiltration in the jejunum. Endoscopic examination revealed melena throughout the rectum, sigmoid, descending, transverse, and ascending colon, while the terminal ileum appeared normal. Scintigraphy with technetium‐99m–labeled red blood cells (Tc‐99m RBCs) detected active but slow gastrointestinal bleeding localized to the territory of the superior mesenteric artery (SMA). Abdominal magnetic resonance angiography (MRA) further identified irregularities in the distal branches of the mesenteric artery within the jejunum. Additionally, endoscopy revealed lymphangiectasia and a hemangioma in the small intestine.

A biopsy obtained endoscopically yielded a 2.5 × 1 × 0.4‐cm fragment of gray‐brownish tissue. Histopathological examination indicated a vascular‐rich lesion with chronic inflammatory cells infiltrating the submucosal and muscular layers and peri‐adipose tissue of the small intestine, suggesting a benign vascular lesion. Immunohistochemical (IHC) staining revealed positive reactions for CD31, CD34, and D2‐40, indicating a lymphatic malformation (lymphangioma).

**Figure 1:**
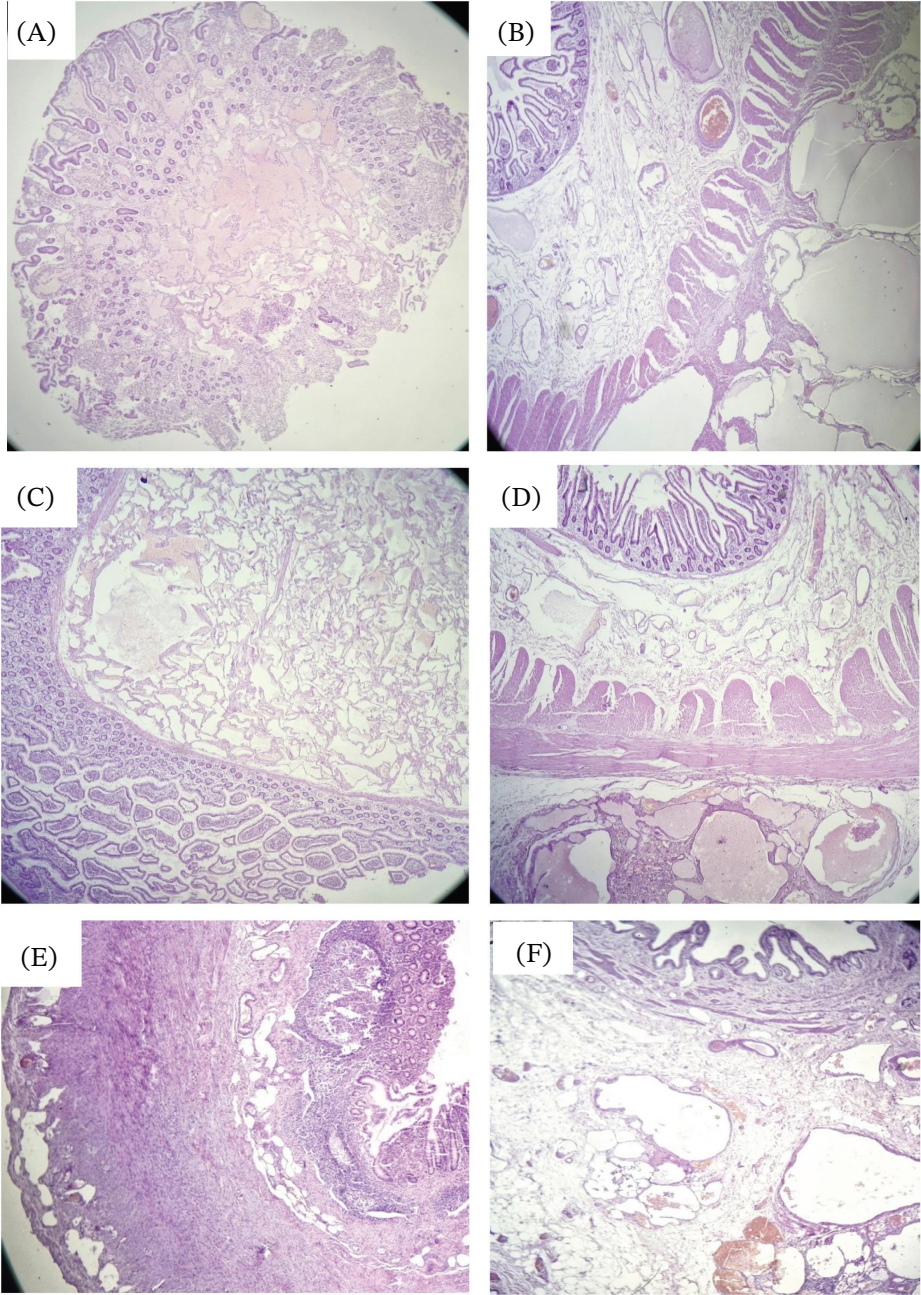
Histopathological findings of lymphangiectasia in various gastrointestinal tissues. Representative photomicrographs showing moderately to severely dilated lymphatic vessels in: (A) submucosa causing a polypoid lesion; (B and C) submucosa and serosa of small intestine; (D) gallbladder wall; (E) appendiceal tissue; and (F) large intestinal wall. The dilated lymphatic vessels appear as empty spaces lined by flattened endothelial cells, displacing normal tissue architecture. (hematoxylin and eosin stain; original magnification: ×40).

Two months later, a second pathology consultation was conducted. The submucosa and muscularis propria microscopically showed cystic spaces lined with a single layer of endothelial cells, which mainly appeared attenuated without atypia. Some vascular spaces contained blood; scattered, dilated lymphatic channels were present in the mucosa, along with patchy lymphoid aggregates (Figure 1). IHC staining showed positive reactions for CD34 and D2‐40 in endothelial cells, with focal CD31 expression. The final diagnosis was a benign vascular tumor, consistent with lymphangioma, with associated diffuse lymphangiectasia.

Due to persistent gastrointestinal bleeding and the need for frequent blood transfusions, the patient was referred to the Abu‐Ali Sina Transplant Center for a small bowel transplant in August 2022. A multidisciplinary team evaluated and listed him for an isolated small intestine transplant. A 21‐year‐old brain‐dead donor, who had sustained fatal injuries in a car accident, was identified. He had stable hemodynamics, matching both blood group and human leukocyte antigen (HLA) during his brief intensive care unit (ICU) stay.

During organ procurement, the SMA and vein (SMV) were isolated using a Cattell–Braasch maneuver, with further exploration of the aorta. The jejunum and ileum were divided using a GIA 75 stapler, and the graft was harvested in the cold phase. Meanwhile, the recipient′s surgery proceeded with a midline incision. The mesenteric vessels were accessed and controlled with a vascular clamp, and the small intestine was resected. A subtotal colectomy was also performed, with the bowel divided using a GIA stapler. Vascular anastomoses were created by connecting the graft SMV to the recipient SMV and the graft SMA to the recipient SMA using 6‐0 polypropylene sutures. Gastrointestinal continuity was re‐established with a side‐to‐side duodenojejunostomy and a distal Bishop–Koop ileosigmoidostomy. A liver biopsy was also taken.

**Figure 2:**
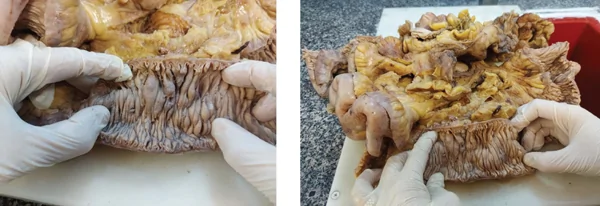
Gross pathology images of the small intestine from a patient with late‐onset intestinal lymphangiectasia show thickened, edematous bowel folds, and markedly dilated lymphatic vessels, indicating extensive lymphatic involvement.

Grossly, the small intestine showed thickened edematous folds with markedly dilated lymphatic channels (Figure 2). Microscopically, there were moderately to severely dilated lymphatic vessels in the mucosa, submucosa, muscularis propria, and serosa of the small intestine (Figure 1A–C). Additional tissue sections demonstrated lymphangiectasia in the large intestine (Figure 1F), appendix (Figure 1E), and gallbladder wall (Figure 1D), indicating multisite gastrointestinal involvement.

All examined mesenteric lymph nodes showed preserved architecture with reactive follicular hyperplasia, with no evidence of lymphoma or other lymphoproliferative disorder.

Postoperative complications began on Day 7 when the patient developed abdominal distension. The evaluation revealed signs of ileus, and a small bowel mucosal biopsy was performed to assess for graft rejection, which was negative. On Day 10, the patient developed a fever, and chest sonography detected pleural effusion. An exploratory laparotomy was performed to exclude peritonitis, but no anastomotic leaks were found. A chest tube was placed on the right side to drain loculated fluid in the right hemithorax, suggesting empyema.

Two days later, the patient developed edema in the left upper extremity. Color Doppler ultrasound indicated more than 50% thrombosis in the proximal left subclavian vein and nearly complete thrombosis in both jugular veins′ proximal and middle segments. Further imaging showed increased intima‐media thickness (IMT) in the left carotid artery, raising concern for atheromatous plaque formation. Despite the initiation of anticoagulation therapy, the patient developed atrial fibrillation. No emboli were observed on echocardiography, but the patient ultimately died a week later due to multiorgan failure.

## 3. Discussion

This case demonstrates intestinal lymphangioma with extensive secondary lymphangiectasia, a critical distinction from PIL. The histopathological diagnosis of lymphangioma, confirmed by positive D2‐40, CD34, and CD31 IHC staining, represents a true lymphatic malformation with neoplastic proliferation of lymphatic tissue rather than purely functional lymphatic obstruction as seen in classic PIL [14, 15]. This pathophysiological distinction is crucial for understanding the disease behavior, prognosis, and management approach.

The definitive histopathological diagnosis in this case was intestinal lymphangioma, a true neoplastic proliferation of lymphatic tissue, with resulting secondary lymphangiectasia, rather than PIL. This distinction is crucial for understanding the clinical presentation, disease behavior, and prognosis.

PIL represents congenital malformation or acquired obstruction of intestinal lymphatic vessels leading to functional dilatation without neoplastic proliferation [14, 15]. In contrast, lymphangioma is a hamartomatous malformation characterized by abnormal proliferation of lymphatic endothelial cells forming cystic spaces [1–3]. Although both conditions result in dilated lymphatic vessels, the underlying pathophysiology differs fundamentally.

The initial imaging findings of mesenteric lymphadenopathy raised concern for lymphoproliferative disorder. However, comprehensive histopathological examination of all resected mesenteric lymph nodes demonstrated only reactive follicular hyperplasia without evidence of lymphoma, atypical lymphoid infiltration, or clonal proliferation. This definitively excluded malignancy and confirmed that the lymphadenopathy represented reactive changes secondary to chronic intestinal inflammation, bacterial translocation, or the underlying lymphatic malformation itself.

The IHC profile with positive D2‐40 (podoplanin), CD31, and CD34 staining confirmed lymphatic endothelial origin and true lymphangioma rather than reactive lymphatic dilatation [16, 17]. This molecular confirmation distinguishes neoplastic lymphangioma from purely obstructive or inflammatory lymphatic changes seen in classic PIL.

PIL, first described by Waldmann in 1961, is characterized by congenital malformation of intestinal lymphatic vessels leading to their dilatation and dysfunction. The classic presentation includes protein‐losing enteropathy manifested by hypoalbuminemia, hypogammaglobulinemia, and lymphopenia, typically appearing in childhood or early adulthood [15]. The pathogenesis involves rupture of dilated lymphatic vessels into the intestinal lumen, causing loss of proteins, lymphocytes, and immunoglobulins [14, 15]. Clinical manifestations include peripheral edema, ascites, pleural effusion, diarrhea, and failure to thrive in children [15].

In contrast, intestinal lymphangioma is a benign hamartomatous malformation characterized by proliferation of lymphatic endothelial cells forming cystic spaces lined by endothelium. Lymphangiomas constitute a relatively rare group of lesions that may manifest with various clinical presentations including cystic masses and, when involving the intestine, malabsorption [1, 2, 18]. Lymphangioma lesions are generally located in the head and neck region and the axilla, rarely developing intra‐abdominally. The most common locations for lymphangiomas of the intra‐abdominal cavity are the mesentery, omentum, mesocolon, and retroperitoneum [3, 4]. Small bowel lymphangioma is a rare benign tumor of the lymphatic system that has been reported as a cause of significant gastrointestinal bleeding [10–13].

Several distinctive features separate this case from typical PIL and warrant detailed discussion. First, the predominant presentation was life‐threatening gastrointestinal bleeding requiring massive transfusion support (more than 20 units of packed red blood cells) rather than the classic triad of hypoalbuminemia, lymphopenia, and hypogammaglobulinemia typically seen in PIL. The patient′s laboratory parameters showed relatively preserved albumin levels and absence of lymphopenia or significant immunoglobulin deficiency [14, 15], contradicting the expected protein‐losing enteropathy pattern. This atypical presentation reflects a distinct pathophysiology: In lymphangioma‐induced secondary lymphangiectasia, mechanical vascular congestion and vessel rupture cause hemorrhage rather than the protein leakage seen in classic PIL.

Second, histopathological examination definitively established lymphangioma as the primary pathological lesion rather than simple lymphatic dilatation. The IHC profile with positive D2‐40 (podoplanin), CD31, and CD34 staining confirmed lymphatic endothelial origin. D2‐40 is a monoclonal antibody to an Mr 40,000 O‐linked sialoglycoprotein and has been demonstrated as a selective marker for lymphatic endothelium [16]. D2‐40 mAb is a selective marker of lymphatic endothelium that reacts with the endothelium of lymphatic channels but does not react with the blood vessel endothelium including arteries, veins, and capillaries [17]. This molecular confirmation distinguishes true lymphangioma from reactive or obstructive lymphatic changes.

Third, histopathological examination of all resected mesenteric lymph nodes revealed normal reactive hyperplasia without evidence of lymphoproliferative disorder, granulomatous inflammation, or metastatic disease. This finding is particularly significant given the preoperative CT imaging demonstrating mesenteric lymphadenopathy. The discrepancy between radiological and pathological findings emphasizes the critical importance of tissue diagnosis in cases with imaging findings suggestive of lymphoproliferative disease. Reactive lymph node hyperplasia in this context likely represents an immune response to chronic intestinal inflammation, bacterial translocation, or the underlying lymphatic malformation itself.

Fourth, the extensive multiorgan involvement affecting not only the entire small intestine and colon but also the appendix and gallbladder represents an unprecedented distribution pattern not previously reported in IL literature. This widespread distribution pattern indicates a more severe form of lymphatic disease with potentially different embryological origins or systemic factors promoting lymphatic malformation development [14].

Our comprehensive literature review of reported IL and lymphangioma cases revealed important epidemiological and clinical patterns (Table 1) [19–33]. Gastrointestinal bleeding as the primary manifestation occurred in only rare reported cases, whereas the vast majority presented with hypoalbuminemia and lymphopenia, the hallmarks of classic protein‐losing enteropathy. Most patients responded to conservative management with medium‐chain triglyceride (MCT) diet, which bypasses intestinal lymphatic absorption by direct portal venous transport. Low‐fat diet in IL has its effect on albumin metabolism [34, 35]. Octreotide has shown efficacy in refractory cases. Treatment with octreotide resulted in a reduction in enteric protein loss, and after 2 weeks of treatment, clinical symptoms as well as hypoproteinemia and hypoalbuminemia gradually became alleviated, though symptoms recurred when octreotide was discontinued [36, 37]. Only one previous case in the literature required surgical intervention, consisting of limited jejunal resection for localized disease. This makes our case the first reported requiring small bowel transplantation for intestinal lymphatic disease and the first reported mortality from this condition.

**Table 1:** Review of 16 cases of extensive intestinal lymphangiectasia [19–33].

Age (years), *n* = 16	
Median (range)	37.5 (19–83)
Mean	40.89 (±18.12)
Sex, *n* = 16	
Male	9 (52.94%)
Female	7 (43.75%)
Diagnostic method, *n* = 17	
Biopsy	14 (82.35%)
Lymphoscintigraphy	2 (11.76%)
Treatment, *n* = 11	
Dietary modification	8 (72.72%)
Jejunal resection + dietary modification	1 (9.09%)
Enteral nutrition + dietary modification	1 (9.09%)
Cyclophosphamide	1 (9.09%)
Time to diagnosis (months), *n* = 14	
Median (range)	24 (0–540)
Mean	121.21 (±179.83)
Follow‐up time (months), *n* = 9	
Median (range)	36 (2–60)
Mean	31.1 ± 20.0
Patient outcome, *n* = 9	
Recovery	7 (77.78%)
Remission	1 (11.11%)
Satisfactory conditions	1 (11.11%)
Lab data, *n* = 16	
Hypoalbuminemia	13 (81.25%)
Hypoproteinemia	7 (43.75%)
Hypogammaglobulinemia	6 (37.50%)
Hypocalcemia	3 (18.75%)
Anemia	5 (31.25%)
Lymphopenia	9 (56.25%)

*Note:* Data compiled from 16 published case reports of extensive intestinal lymphangiectasia in adults (References 19–33, excluding Reference 22 which reported two patients). Not all cases reported complete information for every variable. Cases presenting at initial presentation (*n* = 3) were coded as 0 months for time to diagnosis calculations.

The decision to proceed with small bowel transplantation was based on multiple compelling factors: refractory gastrointestinal bleeding requiring massive transfusion support exceeding 20 units of packed red blood cells, diffuse involvement of the entire small intestine and colon precluding segmental resection, multiorgan gastrointestinal involvement including gallbladder and appendix, and failure of all conservative management strategies. Small bowel transplantation, although traditionally reserved for intestinal failure conditions, has shown improved outcomes with 1‐year actuarial patient survival rates reported in various series [38]. The Intestinal Transplant Registry reported 1‐year patient survival rates ranging from 33% to 87% [38]. Current data indicate that approximately 70%–80% of patients survive for at least 1 year after the procedure [39]. Success rates for intestinal transplant have improved dramatically in recent years, with experienced pediatric centers reporting 1‐year survival rates exceeding 85%, approaching outcomes comparable with liver transplantation [40–42]. However, outcomes vary between centers and patient populations, with adult 1‐year survival rates ranging from approximately 77% to 80% nationally [41].

The fatal outcome from thrombotic and infectious complications warrants careful consideration and analysis. The patient developed extensive venous thrombosis involving subclavian and jugular veins in the early postoperative period, ultimately contributing to mortality. This thrombotic tendency may reflect multiple contributing factors. Surgical tissue injury releases tissue factor, activating the coagulation cascade and promoting hypercoagulability within lymphatic vessels. The outflow obstruction induced by removal of lymphatics draining the arm would generate stasis of lymphovenous channels. The release of Factor X by lymphatic endothelium injury may trigger the coagulation cascade, causing blockage of lymphatic vessels and lymphedema [43]. Patients with chronic conditions may develop persistent inflammation and chronic damage to the lymphatic vessel, leading to loss of lymphatic vessel contraction function, reduced lymphatic drainage, and severe lymphatic thrombosis [43, 44]. The development of empyema likely resulted from bacterial translocation across the compromised intestinal barrier, surgical site contamination, or hematogenous seeding in the context of systemic inflammation and immunosuppression. Progressive multiorgan failure ultimately resulted from overwhelming sepsis, thrombotic complications compromising vital organ perfusion, and systemic inflammatory response syndrome.

This case highlights several important clinical implications for practicing clinicians. First, IL should be included in the differential diagnosis of unexplained adult gastrointestinal bleeding, even in the absence of classic protein‐losing enteropathy features. Second, comprehensive diagnostic evaluation should include advanced endoscopy with careful mucosal inspection, cross‐sectional imaging with CT or MRI to assess disease extent, and tissue biopsy with immunohistochemistry using lymphatic markers (D2‐40, CD31, and CD34) for definitive diagnosis. Third, mesenteric lymphadenopathy identified on imaging requires tissue diagnosis to definitively exclude malignancy or lymphoproliferative disorders. Fourth, assessment for multiorgan involvement may be warranted in severe cases to guide management decisions and surgical planning. Fifth, small bowel transplantation may be considered as a potential therapeutic option for refractory disease with life‐threatening complications, though with full recognition of significant perioperative and long‐term risks. Finally, patients undergoing major surgical procedures or transplantation for lymphatic disorders may require vigilant thrombotic monitoring. Although lymphatic thrombosis occurs in a variety of diseases, it has rarely been reported. For patients with severe, unexplained edema, clinicians may consider the possibility of lymphatic thrombosis. Many cases of lymphatic thrombosis were found in lymphatic biopsy, and further studies are needed to understand the mechanisms and develop novel diagnostic and therapeutic strategies [43].

Future research directions should focus on several key areas to advance understanding and management of severe intestinal lymphatic diseases. First, identifying genetic mutations and molecular biomarkers associated with severe forms of IL may enable risk stratification and predict disease progression. Second, developing targeted therapies that address the underlying lymphatic dysfunction, such as antilymphangiogenic agents or lymphatic flow modulators, could provide alternatives to surgical intervention. Third, establishing evidence‐based guidelines for severe, refractory cases would help standardize management approaches and improve outcomes. Fourth, multicenter collaborative registries could capture rare cases and enable systematic analysis of clinical patterns, treatment responses, and long‐term outcomes. Fifth, investigating the role of perioperative anticoagulation and optimal thromboprophylaxis strategies in patients with lymphatic disorders undergoing major surgery may prevent devastating thrombotic complications.

## 4. Conclusion

We report the first documented case of intestinal lymphangioma with extensive secondary lymphangiectasia manifesting as life‐threatening gastrointestinal hemorrhage in an adult, a presentation unprecedented in the medical literature. The atypical clinical features without classic protein‐losing enteropathy manifestations, multiorgan involvement extending beyond the intestines to include appendix and gallbladder, and normal reactive mesenteric lymph nodes without evidence of lymphoproliferative disease distinguish it fundamentally from classic PIL. This represents the first reported case requiring small bowel transplantation for intestinal lymphatic disease and the first reported mortality from this condition, highlighting both the potential severity of extensive lymphatic malformations and the substantial challenges in managing refractory cases. The fatal outcome from postoperative thrombotic and infectious complications underscores the critical need for careful patient selection, comprehensive preoperative risk assessment, and vigilant postoperative monitoring including thromboprophylaxis strategies. Further research is urgently needed to identify prognostic factors that predict severe disease, establish optimal management algorithms for refractory cases, and develop novel therapeutic approaches targeting the underlying lymphatic dysfunction.

## Funding

No funding was received for this manuscript.

## Conflicts of Interest

The authors declare no conflicts of interest.

## Data Availability

All data relevant to this case report are included in the published article.
